# Genus *Culex* Linnaeus, 1758 (Diptera: Culicidae) as an Important Potential Arbovirus Vector in Brazil: An Integrative Review

**DOI:** 10.3390/life13112179

**Published:** 2023-11-08

**Authors:** Lúcia Aline Moura Reis, Ana Beatriz Oliveira Pampolha, Bruna Lais Sena do Nascimento, Daniel Damous Dias, Pedro Arthur da Silva Araújo, Fábio Silva da Silva, Lucas Henrique da Silva e Silva, Hanna Carolina Farias Reis, Eliana Vieira Pinto da Silva, Joaquim Pinto Nunes Neto

**Affiliations:** 1 Graduate Program in Parasitary Biology in the Amazon Region, Center of Biological and Health Sciences, State University of Pará, Belém 66095-663, Brazil; 2 Institute of Biological Sciences, Faculty of Biological Sciences, Federal University of Pará (UFPA), Belém 66075-110, Brazil; 3 Department of Arbovirology and Hemorrhagic Fevers, Evandro Chagas Institute—IEC/MS/SVSA, Ananindeua 67030-000, Brazil; 4 Graduate Program in Biology of Infectious and Parasitary Agents, Biological Sciences Institute, Federal University of Pará, Belém 66077-830, Brazil

**Keywords:** *Culex*, arbovirus infection, Brazil

## Abstract

The genus *Culex* has 817 species subdivided into 28 subgenera. It has a cosmopolitan distribution, being most abundant in countries with a tropical climate. Understanding the ecology and diversity of viruses circulating in the species of this genus is important for understanding their role as arbovirus vectors in Brazil. To conduct an integrative review to identify the importance of the Culex genus as arbovirus vectors in Brazil. A search was carried out for scientific papers in the PubMed, BVSalud, Patuá-IEC and International Catalogue of Arboviruses: including certain other viruses of vertebrates databases. 36 publications describing arbovirus detections in *Culex* mosquitoes collected in the field in Brazil were evaluated. A total of 42 arbovirus species were detected, as well as studies analyzing the vector competence of *C. quinquefasciatus* for the transmission of four different arboviruses. The study of the *Culex* genus and its role as a vector of arboviruses in Brazil is essential for understanding transmission cycles, with the main aim of reducing cases of human infection. Thus, entomovirological surveillance guides the implementation of actions to detect circulating arboviruses among vectors to anticipate measures aimed at preventing or reducing the risk of arbovirus outbreaks in the country.

## 1. Introduction

The genus *Culex* Linnaeus 1758 currently has 817 valid species and is divided into 28 subgenera according to the Walter Reed Biosystematics Unit (WRBU): *Acalleomyia*, *Acallyntrum*, *Aedinus*, *Afroculex*, *Allimanta*, *Anoedioporpa*, *Barraudius*, *Belkinomyia*, *Carrollia*, *Culex*, *Culiciomyia*, *Eumelanomyia*, *Kitzmilleria*, *Lasioconops*, *Lasiosiphon*, *Lophoceraomyia*, *Maillotia*, *Melanoconion*, *Micraedes*, *Microculex*, *Neoculex*, *Nicaromyia*, *Oculeomyia*, *Phalangomyia*, *Phenacomyia*, *Phytotelmatomyia*, *Sirivanakarnius*, and *Tinolestes*, as well as some species without subgeneric classification [1].

*Culex pipiens* Linnaeus is regarded as the typical species for the Culex genus. According to Forattini (1996) [2], *Culex* adults exhibit minimal morphological variation, with a brown or blackish hue, absence of pre-spiracular and post-spiracular bristles, a well-developed púlvilo, and, with few exceptions, the females of the genus are complex for taxonomic identification due to the high similarity between species of the same subgenus, with species often being subdivided into groups, subgroups, and complexes. Thus, the study of male genital morphology provides a more reliable method for accurate taxonomic identification of species [2,3,4].

In recent decades, molecular characterization tools have been implemented for taxonomic identification and phylogenetic reconstruction studies due to difficulties in morphologically identifying species in the genus. The mitochondrial gene cytochrome oxidase subunit I (COI), also known as the DNA barcode, is used as the primary target to determine evolutionary relationships between species [5,6,7].

The immature forms of the species of the genus may be found in nature in various water accumulations from small leaves, bracts, pools of water on the ground, lakes, irrigation fields and similar. Domestic environments also provide a variety of larval habitats such as ditches, gutters, or any environment rich in organic matter [8,9,10,11].

One of the most epidemiologically important subgroups is the *pipiens* subgroup, made up of the species *C*. *australicus* Dobrotworsky & Drummond, *C*. *globocoxitus* Dobrotworsky, *C*. *pallens* Coquillett, *C*. *pipiens* Linnaeus, *C*. *quinquefasciatus* Say [12,13], that are highly morphologically similar but differ in behavior, feeding, mating, oviposition, and geographic distribution [14,15,16].

Among the behavioral differences between the species is the female reproductive process, which can take two forms: autogenous and anautogenous. Autogeny consists in the ability of some species to make one or more first ovipositions without blood repast. Anautogeny, on the other hand, is characterized by the necessity of feeding before the first oviposition, which is considered the most common among species of the family *Culicidae*. In theory, autogenous females have a lower initial risk of infection from blood feeding. However, anautogenous females have a higher risk of acting as a vector species from the beginning of the winged phase due to the greater likelihood of contact with viremic hosts [10,15].

Among the epidemiologically significant species of the *Culex* genus, *C*. *quinquefasciatus* is noteworthy. This species is highly adapted to urban environments where it can find favorable habitats for the development of its immature forms. It is a species that is primarily nocturnal, with a preference for indoor environments and endophagous feeding habits [15,16,17].

In a study conducted by Garcia-Rejon et al. (2010) [18], the species *C*. *quinquefasciatus* showed a greater preference for blood meal in galliform birds such as roosters, hens, and turkeys, as well as passerines such as songbirds, pintails, and sparrows, and columbiform birds such as doves and turtle doves. Among mammals, dogs and humans are the main sources of blood feeding, since these mosquitoes are highly adapted to breeding in urban and domestic environments [18,19]. These feeding patterns are related to the transmission cycles of the arboviruses, as these vertebrates act as amplifiers during the viremic period and as a source of blood meals for invertebrates, which in turn act as vectors and reservoirs, transmitting the virus to other susceptible vertebrate hosts, such as humans [19,20,21].

Thus, this study aims to conduct an integrative review of the literature in databases and in the International Catalog of Arboviruses, aiming to identify the importance of mosquitoes of the genus *Culex* as vectors of arboviruses in Brazil, analyzing the viruses already detected in the genus, either by viral isolation techniques or by molecular biology analyses.

## 2. Materials and Methods

### 2.1. Concept of the Study

This study Consists of a Descriptive Integrative Review of the Literature.

The integrative review consists of a large bibliographic analysis methodology that aims to synthesize the knowledge generated in a given field, bringing together the main results of both experimental and epidemiological research. It seeks to critically and carefully synthesize the knowledge generated with a view to incorporating the results of research into the practice of science, since summarizing the results of different studies on a given topic in a single study allows other researchers to identify unanswered questions and gaps in knowledge, as well as encouraging the creation of new research problems [22,23].

The integrative review is comprised of six stages: determination of the topic to be addressed; definition of inclusion and exclusion criteria; selection of studies based on the analysis of descriptors, titles, and abstracts; construction of a database containing information such as the population, methodological design, and main results; analysis and interpretation of the results; and presentation of the findings [24,25].

### 2.2. Search Strategies

The papers were searched in the National Library of Medicine (PubMed), the Regional Portal of the Virtual Health Library (BVSalud), the Digital Repository of the Evandro Chagas Institute (Patuá), and the International Catalogue of Arboviruses: including certain other viruses of vertebrates [26,27].

**Figure 1:**
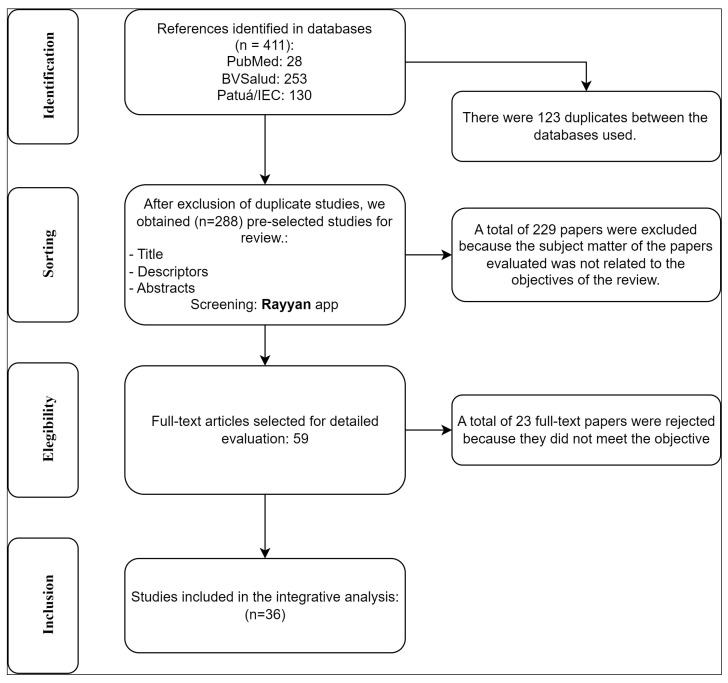
Flowchart of the process of identification, selection, eligibility, and inclusion of the scientific articles used in the review-2023.

The following descriptors were used to conduct the search: *Culex*, arbovirus infections, arbovirus, disease vectors, and Brazil, defined by Medical Subject Headings (MeSH). Only “AND” was used as a Boolean operator, since we were looking for papers that integrated the topics covered by all the selected descriptors. The combinations used were “*Culex*” AND “arbovirus infections” AND “Brazil”, “*Culex*” AND “arbovirus” AND “Brazil”, and “*Culex*” AND “disease vectors” AND “Brazil” (Figure 1).

Based on the search strategies, an initial total of 405 papers were obtained. The search results were saved in “.ris” and “.txt” files for sorting using the Rayyan Systematic Review platform: https://www.rayyan.ai/ (accessed on 11 July 2023) [28].

### 2.3. Inclusion and Exclusion Criteria

Inclusion criteria included papers that investigated *Culex* mosquitoes as arbovirus vectors in Brazil, providing data on virus species identification from field samples, in vivo and in vitro experimental testing of vector competence of Culex species, and exploring the potential involvement of the genus in arbovirus transmission cycles in the country. In addition to articles and M.Sc. theses, we also accepted Ph.D. theses and book chapters, as well as works written in Portuguese, English and Spanish, with full-text availability both on private Internet networks and on the institutional networks of the State University of Pará (UEPA) and the Evandro Chagas Institute (IEC). It was decided not to specify a particular time frame since numerous viral identification studies have been published for over a decade.

Exclusion criteria were papers that did not discuss the genus of interest in their results, papers that only mentioned *Culex* species in the text and did not present data of interest to the research, papers whose main topic was insecticide resistance, and duplicate papers.

## 3. Results

**Figure 2:**
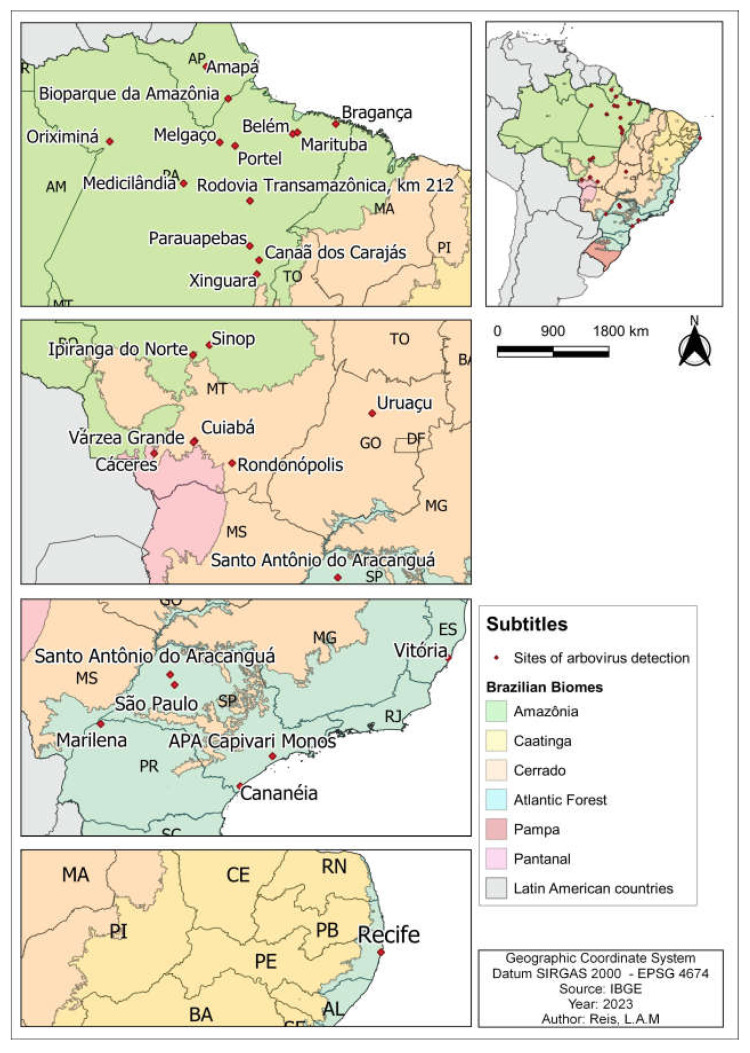
Map of Brazil showing arbovirus detection sites in Genus *Culex* mosquitoes, 2023.

A total of 36 papers were evaluated, and data from the International Catalogue of Arboviruses: including certain other viruses of vertebrates by Karabatsos [27] and the Centers for Disease Control and Prevention [26], which report the detection of arboviruses in different species of mosquitoes of the Culex genus (Table 1), collected in various locations in Brazil (Figure 2).

Eight studies were analyzed to evaluate the ability of the Culex quinquefasciatus species to transmit the arboviruses that have been detected in this species when collected in the field. The experiments were conducted with Zika, Oropouche, Mayaro and West Nile viruses using mosquito colsonies from different cities in Brazil (Table 2).

From the presented data, a total of 44 arboviruses were identified in mosquitoes of the *Culex* genus. These viruses were classified into seven viral families and seven genera, as well as one ungrouped virus (Table 3). Of the total number of arboviruses identified, 23 have caused infections in humans, ranging from mild symptoms to more severe conditions such as encephalitis and coma.

## 4. Discussion

### 4.1. Togaviridae: Alphavirus

#### 4.1.1. *Aura Virus* (AURAV)

The Aura virus was first isolated in 1959 from mosquitoes of the subgenus *Culex* (*Melanoconion*) sp. collected at the Instituto de Pesquisa e Experimentação Agropecuária do Norte (IPEAN), now known as EMBRAPA-Amazônia Oriental, using the human attraction method to collect and the mouse viral isolation methodologies for viral detection [26,27,33]. Woodall [56] points out that in Brazil, the Aura virus has only been isolated from mosquito samples.

AURAV is a positive-sense, single-stranded RNA virus belonging to group A and is serologically related to Sindbis and *Western Equine Encephalitis viruses* (WEE), thus making up the WEE-Sindbis complex [63]. It exhibits low pathogenicity in mice. Viral titers varied between 8.3 and 8.5 log LD50c only after the virus had been passaged eight times in mouse brains. Histopathological analyses revealed the occurrence of encephalitis and focal myocardial degeneration, and Hemagglutination Inhibition (HI) tests indicated the presence of antibodies, in low titers, also in samples from marsupials, rodents and horses [26,27,33].

#### 4.1.2. *Caaingua Virus* (CAAV)

In 2017, the Fiocruz-PR Reference Laboratory for Emerging Viruses (ViroMol) received 62 serum samples from patients in Marilena, Paraná (PR) who were presenting symptoms of fever, myalgia, headache, low back pain, retroorbital pain and arthralgia. All samples tested negative for Mayaro virus (MAYV), Oropouche virus (OROV) and West Nile virus (WNV). During the outbreak period, researchers collected samples of Culex mosquitoes in the municipality and identified a new Alphavirus called Caaingua virus. Based on nucleotide alignment, it was found to have a 74% similarity to the Venezuelan Equine Encephalitis (VEEV), Western Equine Encephalitis (WEEV), and Eastern Equine Encephalitis (EEEV) viruses [54].

#### 4.1.3. *Chikungunya Virus* (CHIKV)

CHIKV is a positive-sense, single-stranded RNA virus that belongs to the Semliki Forest antigenic group. Its genome is closely related to that of O’nyong nyong virus (ONNV) [64,65]. The first isolation occurred in 1954 after an outbreak of a disease with symptoms similar to dengue between 1952 and 1953, in the province of Tanganyika, now called Tanzania [65]. Its enzootic transmission cycle mainly occurs between *Aedes* mosquitoes as the primary vectors and non-human primates as reservoirs and amplification hosts [34,64,65].

Regarding the phylogenetic analysis of CHIKV, there are presently four acknowledged genotypes designated based on their geographical location: genotype I from West Africa, genotype II from East Central South Africa (ECSA), genotype III from Asia, and genotype IV from the Indian Ocean (IOL) [34,66].

In Brazil, Ferreira et al. [36] isolated in C6/36 cells CHIKV in seven *Cx*. *quinquefasciatus* pools collected from the cities of Cáceres and Cuiabá (MT), and the presence of co-infection between CHIKV and MAYV viruses in two of these pools, both from the city of Cuiabá. Neves et al. [45] detected CHIKV in 42 pools of *Cx*. *quinquefasciatus* mosquitoes collected from different locations in the cities of Cuiabá and Várzea Grande, including forest parks, the campus of the Federal University of Mato Grosso (UFMT), and human dwellings, using RNA extraction and RT-PCR for arboviruses, and isolation in C6/36 cells.

Krokovsky et al. [41], in addition, identified CHIKV from a pool containing engorged *Cx*. *quinquefasciatus* females collected at the São Lourenço da Mata UPA in the city of Recife (PE).

Cruz et al. [34] detected CHIKV in two pools of female *Cx*. *quinquefasciatus* mosquitoes collected in Xinguara, Pará, using viral isolation and RT-qPCR. The Ct values were 28.2 and 33.7. The phylogenetic analysis indicated that the strains belong to Genotype II, East Central South Africa (ECSA), and share a close resemblance with other strains found in North and Northeast Brazil.

#### 4.1.4. *Eastern Equine Encephalitis Virus* (EEEV)

Eastern equine encephalitis virus (EEEV) is a neurotropic, non-segmented RNA arbovirus responsible for several epidemics and epizootics in horses and pheasants in the United States. EEEV was first isolated in 1931 in Delaware, USA, from the brains of horses exhibiting symptoms of incoordination, fever, hyperirritability, convulsions, and eventually death [27]. Its natural cycle includes passerine birds as the main amplification hosts and mosquitoes, mainly of the *Culiseta melanura* species, as vectors [67,68].

In Brazil, epizootic events in horses have been reported in the state of Pará, specifically in the city of Bragança in 1960 [69]. Additionally, isolates from mosquitoes belonging to the *Culex* spp. and *Cx*. (*Mel*.) *taeniopus* were collected from sentinel animals in the forest of the Instituto de Pesquisa e Experimentação Agropecuária do Norte (IPEAN), currently known as EMBRAPA-Amazônia Oriental, in the city of Belém in 1963. The species Cx. (*Melanoconion*) *taeniopus* was found to be the most prevalent [56,70]. Karabatsos [27] and Woodall [56] also reported detecting EEEV in Brazil, in samples of *Cx*. *restuans*, *Cx*. *salinarius*, *Cx*. *quinque*-*salinarius*, *Cx*. *nigripalpus*, and *Cx*. *taeniopus*. Segura et al. [49] isolated EEEV from *Cx*. (*Mel*.) sp. collected in Portel (PA) in 2004.

#### 4.1.5. *Mayaro Virus* (MAYV)

Originally identified in serum samples from forest workers in Trinidad in 1954, MAYV is a single-stranded RNA arbovirus. It is mainly transmitted in the wild by mosquitoes of the species *Haemagogus janthinomys* and the non-human primates (NHP) are the main amplification hosts [48,60,71,72].

MAYV is classified under serogroup A, belongs to the Semliki Forest complex and shares significant antigenic similarity with the Chikungunya, Sindbis, and Ross River viruses [73]. The virus has three lineages: disseminated (D), limited (L), and new (N), as determined by phylogenetic analysis [71]. Note that Uma virus is a subtype of MAYV [27].

In a study conducted by Ferreira et al. [36], MAYV was isolated in pools of *Cx*. *quinquefasciatus* mosquitoes collected from the cities of Cáceres, Cuiabá, Rondonópolis and Sinop in the state of Mato Grosso, with the circulation of genotype D of the virus detected in male individuals of the same species. Neves et al. [45] identified MAYV in samples of male and swollen female *Cx*. *quinquefasciatus* mosquitoes from various locations in the city of Cuiabá (MT), including forest parks and human dwellings, and coinfection with Mayaro and Chikungunya viruses in four *Cx*. *quinquefasciatus* pools.

Serra et al. [50] identified the presence of MAYV in a pool of *Cx*. *quinquefasciatus* mosquitoes collected from different regions of the city of Cuiabá (MT) using virus isolation and RT-PCR. The co-infection between MAYV and Dengue virus (DENV-4) was also detected in five pools analyzed, five of which were also positive for DENV-4. Phylogenetic analysis of the isolates showed similarity with other sequences obtained from human samples taken from the cities of Cuiabá and Várzea Grande (MT), as well as with sequences belonging to the L genotype of ticks of the genus *Ixodes* spp., from *Haemagogus janthinomys* mosquitoes, and from humans from the state of Pará (PA).

Karabatsos [27] also reports the identification of MAYV in *Culex* sp. samples collected from the forest belonging to the Instituto de Pesquisa e Experimentação Agropecuária do Norte (IPEAN), currently EMBRAPA-Eastern Amazon, in Belém (PA). However, the year of identification is not described.

Regarding vector competence studies, Krokovsky et al. [60] conducted experiments using a population of *Cx. quinquefasciatus* (CqSLab) collected in Recife (PE). The population was artificially fed with blood infected with the MAYV/BR/Sinop/H307/2015 (MH513597) strain derived from human serum, which was cultivated in VERO cells and had a titer of 10^8^ PFU/mL. The engorged females were kept for a period of 14 days post-infection (dpi), in which on the 3rd, 7th and 14th dpi 15 females were collected on each dpi and the presence of MAYV was assessed by RT-qPCR in the midgut (MID), for analysis of the infection rate (IR), and salivary gland (SG) regions, for the dissemination rate (DR), obtaining an IR of only 14.28% on the 3rd dpi, 13.1% on the 7th and 14.81% on the 14th dpi, but despite the low number of positive midgut samples, 10 positive salivary glands were detected.

The researchers [60] also employed animal models to evaluate if the viral load identified in mosquitoes can effectively infect naive mice and subsequently infect new susceptible mosquitoes, ultimately confirming the complete transmission cycle. To do this, the IFNAR BL/6 (-Ifnar1^tm1.2Ees^/J) mouse strain was used, with eight mice used as the test group and four as the control group, and it was observed that none of the mice in the infection group showed signs of infection (isolation, lethargy, curved posture, weight loss, paw edema and diarrhea) after being fed blood from infected *Cx*. *quinquefasciatus* mosquitoes (CqSLab), and brain, liver and gonad samples were analyzed by RT-qPCR, and out of a total of 24 tissue samples, only one brain was positive for MAYV with a Cq of 32.9. On the 7th dpi, these mice were utilized as a blood source for virgin *Cx. quinquefasciatus* females to conduct a blood meal. Subsequently, all the females were gathered and subjected to RT-qPCR analysis of the whole body on the 7th dpi to ensure absence of positive results.

Vector competence studies were also performed by Pereira et al. [61], in which *Cx*. *quinquefasciatus* mosquitoes from Belo Horizonte (MG) were artificially fed with human blood infected with MAYV (unspecified strain). The experiments contained different viral titers, 10^9^ PFU/mL in the first and 6 × 10^6^ PFU/mL in the second. Infection and transmission rates were examined on the 7th and 14th dpi through head and thorax, as well as saliva analysis. The results showed that only two females tested positive for MAYV, resulting in a low infection rate of 2.5%. Additionally, no positive saliva samples were found.

#### 4.1.6. *Mucambo Virus* (MUCV)

MUCV is a segmented RNA virus that belongs to the Venezuelan Equine Encephalitis (VEE) complex. The complex comprises six serotypes, named from I to IV, with MUCV belonging to subtype IIIA of group A of the Alphaviruses [66,74].

MUCV was first isolated in 1954 from serum and plasma samples of a male *Cebus apella* captured in the Oriboca forest area, now called the Metrópole da Amazônia Wildlife Refuge, located in the city of Marituba (PA). Also been identified in pools of *Culex* sp. (B7, B9 and B19), *Culex* spp., *Cx*. *portesi* and *Cx*. (*Mel*.) sp. mosquitoes collected in the city of Belém [27,38,56].

Karabatsos [27] reports that in an experimental study conducted with naturally infected *Cx*. *portesi* species, transmission of MUCV to susceptible mice was observed.

### 4.2. Flaviviridae: Orthoflavivirus

#### 4.2.1. Bussuquara Virus (BSQV): *Orthoflavivirus aroaense*

BSQV was first isolated in 1956 from two blood samples collected from the non-human primate *Alouatta beelzebul* in the forest of the Instituto Agronômico do Norte (IAN), now EMBRAPA-Amazônia Oriental [32]. It is a flavivirus found in the wilds of Brazil that primarily induces acute febrile illnesses. It is classified serologically as part of the Japanese encephalitis virus (JEV) complex [75].

Causey et al. [32] reported that no new cases of BSQV detection were found from its first isolation in 1956 until 1959. However, in May 1959, eight new strains of the virus were identified, one of which had been isolated from a pool containing 51 *Cx*. (*Melanoconion*) sp. mosquitoes captured in the forest of the Instituto Agronômico do Norte (IAN). Pinheiro [48], in describing arboviruses in the Amazon, reported detecting BSQV in *Culex* sp. B1 (B22) and *Culex* sp. in the city of Belém.

Karabatsos [27], Woodall [56] and Hervé et al. [38] also reported the identification of BSQV in *Culex* sp., *Cx*. (*Mel*.) sp., *Cx*. (*Mel*.) *taeniopus*, *Cx*. *vomerifer*, *Culex* sp. B7 and *Culex* sp. B1 collected from the State of Pará, highlighting that in experimental studies carried out with *Cx. quinquefasciatus*, parentally inoculated, showed high virus titers in the salivary gland and *Culex* sp. B1 mosquitoes were competent to transmit BSQV to mice.

In an entomological surveillance study carried out by Araújo et al. [29] the viral genome of BSQV was also detected in a pool of *Cx*. (*Mel*.) *portesi* captured in Caxiuanã National Forest using the MiniSeq (Illumina, Santiago, CA, USA) platform, showing 94% of homology.

#### 4.2.2. Dengue Virus (DENV): *Orthoflavivirus denguei*

DENV is an arbovirus that consists of four serotypes, DENV 1 to 4, each with unique antigenic characteristics. The main vectors of this virus in urban areas are *Aedes aegypti* mosquitoes, with the potential involvement of the *Aedes albopictus* species currently under investigation [76,77].

Krokovsky et al. [41] conducted a study in Recife (PE) from 2015 to 2017, where they found co-infection between DENV2 and ZIKV serotype II in four pools of *Cx*. *quinquefasciatus*, as well as DENV2 and DENV4 in a single pool of *Cx. quinquefasciatus* and DENV2 and DENV3 in another pool of *Cx. quinquefasciatus*. The authors also detected DENV in four pools of *Cx. quinquefasciatus* in 2015.

Barrio-Nuevo et al. [31] detected DENV serotype 2 in pools of *Culex* spp. and *Cx*. *vaxus* collected in the Capivari-Monos Environmental Protection Area, located in the city of Sao Paulo, Brazil. The techniques of virus isolation in C6/36 strains and indirect immunofluorescence assay (IFA) were used for detection. The IFA-positive samples were then subjected to qRT-PCR using flavivirus genus-specific primers.

DENV serotype 4 was detected in *Culex* mosquito samples collected in the city of Cuiabá, MT. In studies conducted by Moraes et al. [42] and Serra et al. [50], 105 pools of *Cx*. *quinquefasciatus* and two of *Cx*. *bidens*/*interfor* were found to be positive for DENV4. Notably, the two pools of *Cx*. *quinquefasciatus* were composed of non-engorged females.

#### 4.2.3. Ilhéus Virus (ILHV): *Orthoflavivirus ilheusense*

ILHV was first isolated in 1944 from pools containing mosquitoes of the *Aedes* and *Psorophora* genera collected at Fazenda Parataquisse, near the city of Ilhéus, Bahia. Its enzootic transmission cycle is mainly maintained between mosquitoes, as ILHV has been identified in several species, including *Cx*. *coronator*, *Haemagogus spegazzinii*, *Sabethes chloropterus*, and *Psorophora ferox*, as well as in wild birds, such as *Sporophila caerulescens* and *Florida caerulea* [35,78,79].

Cunha et al. [35] reported the isolation of ILHV in a group of *Culex* spp. collected in the city of Santo Antônio do Aracanguá (SP) in 1994, using the Pan-Flavivirus qRT-PCR assay for NS5-3`NCR gene for viral detection and the HiSeq 2500 Sequencer (Illumina, Santiago, CA, USA) for nucleotide sequencing, while Araújo et al. [29] detected the ILHV viral genome in samples of *Cx*. *portesi* collected in the Caxiuanã National Forest (PA) using the MiniSeq platform (Illumina, Santiago, CA, USA), which shows a homology of 92%.

Vieira et al. [55] identified ILHV through RT-PCR in two pools of female *Culex* mosquitoes but failed to isolate the virus in cell culture. One positive pool comprised *Cx*. *coronator* collected in Sinop (MT) in 2015, while the other pool included *Cx*. (*Mel*.) sp. collected in Ipiranga do Norte (MT) in 2016.

Karabatsos [27] reports that in experimental studies carried out with the species *Cx*. *fatigans*, it was observed that it was a competent vector for transmitting ILHV to mice and chickens.

#### 4.2.4. Rocio Virus (ROCV): *Orthoflavivirus ilheusense*

ROCV is a neurotropic virus with single-stranded, positive-sense RNA. It was first isolated in Brazil in 1970 during an outbreak of meningoencephalitis in the state of São Paulo (SP). This virus belongs to the Ntaya virus group, which includes other viruses like Bagaza virus and Ilheus virus [75,80,81].

The transmission cycle of the virus remains undefined, but it is hypothesized to persist among vectors, mainly *Psorophora ferox* and *Aedes scapularis*, and amplification hosts, such as the *Zonotrichia capensis* species of migratory birds [81].

In a study conducted by Araújo et al. [29] in the Caxiuanã National Forest (PA), the viral genome of ROCV was identified in a pool of *Cx*. (*Mel*.) *portesi* captured using the CDC trap method in the soil region, by investigating the viral genome and nucleotide sequencing using the MiniSeq (Illumina, Santiago, CA, USA) platform, showing 92% of homology, highlighting the need to conduct studies to elucidate the role of this species in transmission cycles.

#### 4.2.5. St. Louis Encephalitis Virus (SLEV): *Orthoflavivirus louisense*

The SLEV arbovirus is a member of the Japanese encephalitis virus complex. Its first isolation in Brazil occurred in 1960 from a mosquito pool of *Sabethes belisarioi* species from Pará state [39]. *Culex* mosquitoes are the main vector in its enzootic cycle, while birds act as amplifying hosts [82].

Currently classified into eight lineages and 15 subtypes, these lineages are related to the geographical distribution of the virus, with Brazilian strains belonging to genotypes II, III, V and VIII [39,82].

Travassos da Rosa et al. [53] reported the identification of SLEV in the Amazon Region during different years. The virus was isolated in 1964 from *Cx. declarator* collected in forests adjacent to the city of Belém (PA). Additionally, SLEV was isolated between 1968 and 1969 from a pool of *Cx. coronator* captured in the forest of the Guamá Ecological Research Area (APEG), contiguous to the city of Belém, currently belonging to EMBRAPA-Amazônia Oriental. In 1970, SLEV was isolated from various species including *Cx. declarator*, *Cx*. (*Mel*.) *spissipes*, and *Cx*. *coronator*. Subsequently, its isolation from *Cx*. *declarator* mosquitoes continued between 1971 and 1972, while during 1973 and 1974, SELV was also isolated from *Cx*. *declarator* mosquitoes, *Cx*. *coronator*, *Culex* (*Mel*.) *portesi*, *Culex* sp. *morphospecies B19*, and *Culex* (*Cux.*) sp. in Belém (PA).

Segura et al. [49] additionally reported the detection of SLEV in *Cx. declarator* collected in 2002 in the city of Medicilância (PA). Heinen et al. [39] detected SLEV in a pool that included a female of the *Cx. quinquefasciatus* species collected in the Bela Vista neighborhood of the city of Cuiabá (MT) in 2013, with a minimum infection rate (MIR) of 0.29 per 1000 species, and phylogenetic analysis revealed that the isolated strain belonged to the VA genotype of SLEV, also identified in animals from the state of Pará.

Serra et al. [50] isolated SLEV from a pool containing a non-engorged female *Cx. quinquefasciatus* collected in the central west of Cuiabá, and phylogenetic analysis also indicated that this strain belonged to the VA subgenotype and showed similarity to other strains isolated from human samples identified in 2012.

#### 4.2.6. West Nile Virus (WNV): *Orthoflavivirus nilense*

WNV is an arbovirus that affects the nervous system and was first isolated in 1937 from a human sample taken in the West Nile district of Uganda. Mosquitoes of the *Culex* genus are mainly responsible for transmitting the virus, and wild birds are the primary amplification hosts. In 2018, the virus was first isolated in Brazil from an equine sample collected in the state of Espírito Santo (ES) [44,83].

In Brazil, the first detection of WNV in arthropods was reported by Nunes Neto et al. [44] where the virus was isolated from a pool of 40 specimens of *Cx*. (*Mel*.) sp. mosquitoes collected in 2017 in Canaã dos Carajás, Pará (PA), and identified by both viral isolation in cell culture and RT-qPCR, with a Ct value of 9.69. The strain phylogenetic analysis demonstrated similarity to other isolates from Brazil, obtained from animals like horses. The strain is phylogenetically closer to Espírito Santo strains, and it is classified in lineage 1A of WNV.

Regarding experimental studies analyzing the vectorial competence of *Culex* mosquitoes in transmitting strains isolated in Brazil, Reis et al. [62] conducted studies using colonies of *Cx. quinquefasciatus* from Ananindeua, PA, using F1 and F3 generations as well as the BEAN854747 WNV strain (GenBank: MH643887) isolated from an equine sample from São Mateus, ES. The infection rate was assessed using body samples (thorax + abdomen), while head samples were analyzed to determine the dissemination rate and saliva samples were evaluated to determine the transmission rate. These assessments were conducted on the 7th, 14th, and 21st dpi. *Cx. quinquefasciatus* exhibited a significantly high susceptibility to WNV infection, with an infection rate of 92% on the 7th dpi, 98% on the 14th dpi, and 100% on the 21st dpi. Saliva samples also tested positive for the virus on all three dpi’s, resulting in a transmission rate of 4% on the 7th and 14th dpi, and 48% on the 21st dpi.

#### 4.2.7. Zika Virus (ZIKV): *Orthoflavivirus zikaense*

ZIKV is a positive-sense RNA virus first isolated in 1947 from a sample of rhesus monkeys and in 1948 from *Aedes africanus* [37]. Its primary vector is the *Aedes aegypti* mosquito [30,84].

Ayres et al. [30] isolated ZIKV from two pools of non-engorged *Cx. quinquefasciatus* females collected in Vitória (ES) in 2016, using VERO cell culture (CCL-81, Cercopithecus aethiops) and phylogenetic analysis showed that the ZIKV/Cx.quinquefasciatus/Brazil/ES24/2016 (Cxq_ES24) genome is related to other strains detected in Haiti in 2014 and in the state of Pernambuco in 2015 during a ZIKV outbreak.

Ferreira et al. [36] and Neves et al. [45] both isolated ZIKV in *Cx. quinquefasciatus* pools collected in the state of Mato Grosso during 2017–2018. Specifically, Ferreira et al. identified ZIKV in two pools from the cities of Cuiabá and Sinop, while Neves et al. found ZIKV in five pools from various state parks and residential areas in the city of Cuiabá, including one pool composed of male specimens and another of non-engorged females. Neves et al. [45] also identified mosquitoes of this species co-infected with both Zika virus and Chikungunya virus.

Krokovsky et al. [40,41] identified multiple pools of *Cx. quinquefasciatus* that tested positive for ZIKV in Recife (PE) between 2016 and 2017, and detected ZIKV in pools of Cx. *quinquefasciatus* collected in 2018, which the pools were divided into engorged and non-engorged females, isolating the virus in both groups.

Guedes et al. [37] collected *Cx. quinquefasciatus* in Recife (PE) in 2016, in which non-engorged females were analyzed by RT-qPCR, identifying three pools positive for ZIKV.

Paiva et al. [46] conducted multiple collections between 2015 and 2017 in areas near healthcare centers and human dwellings in Recife (PE) and identified the ZIKV genome in three pools of *Cx. quinquefasciatus*, only one of which contained females with signs of engorgement, and the ZIKV genome was also detected in a pool containing males.

Due to the detection of ZIKV in mosquitoes of the genus *Culex* collected in the field, several vector competence studies have been conducted to determine the importance of this vector in the urban transmission cycle of ZIKV.

Fernandes et al. [57] utilized colonies of *Cx. quinquefasciatus* from four neighborhoods located within the city of Rio de Janeiro (RJ): Manguinhos, Triagem, Copacabana, and Jacarépagua. The colonies were infected with two strains of ZIKV (Rio-U1 and Rio-S1). The virus titer measured 10^6^ PFU/mL. Females that were engorged were assessed on the 7th, 14th, and 21st dpi and the infection rate (IR) was assessed using processed thorax and abdomen, the dissemination rate (DR) was determined using the head, and the transmission rate (TR) was assessed using the saliva. The findings revealed that the *Cx. quinquefasciatus* colonies had almost no IR, with only one specimen out of 30 evaluated, fed with the ZIKV Rio-U1 strain, being positive at 14th dpi, with a viral titer of 7 PFU/mL and 1814 RNA copies/mL. There were no positive results for specimens in the evaluation of DR and TR, regardless of the analyzed dpi. Thus, it was estimated that under laboratory conditions, *Cx. quinquefasciatus* were not competent vectors for transmitting ZIKV.

Fernandes et al. [58] conducted studies on *Cx. quinquefasciatus* collected in Recife (PE) and Campina Grande (PB), areas with high incidences of microcephaly cases linked to ZIKV. The researchers performed artificial infections using three strains from Recife (PE), Sumaré (SP), and Rio de Janeiro (RJ), with viral titers of 2.3 × 10^6^ PFU/mL, 1.68 × 10^7^ PFU/mL, and 3.55 × 10^6^ PFU/mL, respectively. F1 generation mosquitoes were evaluated for body, head, and saliva samples on the 7th, 14th, and 21st dpi. The findings revealed that the species was refractory towards ZIKV, independent of the viral strain used, with only one sample from the Recife colony showing weak body positivity for the Rio de Janeiro strain, although the virus did not spread.

Guedes et al. [37] utilized *Cx. quinquefasciatus* mosquitoes from Recife (PE), which were collected in the Peixinhos neighborhood in 2009. For artificial infection, the researchers used ZIKV virus with a titer of 10^6^ PFU/mL. Female mosquitoes were dissected on the 3rd, 7th, and 15th dpi to obtain midgut and salivary gland samples. The results showed that only on the 3rd dpi were no ZIKV-positive samples identified, and viral copies were identified in the salivary glands by RT-qPCR with Ct of 37.6, 38 and 38.5.

### 4.3. Peribunyaviridae: Orthobunyavirus

#### 4.3.1. Ananindeua Virus (ANUV): *Orthobunyavirus ananindeuaense*

ANUV was first isolated in 1966 from a blood sample of a male marsupial (*Caluromys philander*) captured in the Utinga Forest, located in the city of Belém (PA) [26]. According to the results of the complement fixation (CF), hemagglutination inhibition (HI), and neutralization (N) tests, ANUV is a member of the Guamá group [26,27].

According to reports by Travassos da Rosa et al. [85], Pinheiro [48], and The International Catalog of Arboviruses [26], ANUV has been isolated in several mosquito species, mainly in the city of Belém (PA) in the genus *Culex*, such as *Cx*. (*Mel*.) sp., *Culex* sp. B19 and B27, *Cx*. (*Mel*.) *portesi*, *Cx*. *taeniopus* and *Cx*. *vomerifer*. Hervé et al. [38] also detected ANUV in *Cx. pedroi* and *Cx. aikenii*.

#### 4.3.2. Acará Virus (ACAV): *Orthobunyavirus acaraense*

ACAV was isolated in 1961 from a Swiss mouse’s brain and liver sample. The mouse was around 3 days old and was captured at the Instituto Agronômico do Norte (IAN), EMBRAPA-Amazônia Oriental. According to The International Catalog of Arboviruses [26], it is similar to members of the Capim group.

Karabatsos [27] and Hervé et al. [38] reported the detection of ACAV in *Culex* mosquitoes in Pará state. In experimental studies with parentally inoculated *Cx. quinquefasciatus*, ACAV was identified in the salivary glands after four serial passages.

#### 4.3.3. Apeú Virus (APEUV): *Orthobunyavirus apeuense*

APEUV was first isolated in 1955 from serum and plasma samples of the sentinel NHP *Cebus apella*, captured in the Oriboca forest in the state of Pará. Upon analysis using the HI and N tests, its close relation to the Caraparu virus was revealed, as well as its relation to the Marituba virus through the CF test. Thus, APEUV was labeled as a unique virus type that belongs to the Caraparu complex [26].

Regarding its detection in mosquitoes of the *Culex* genus, the virus has only been found in samples of *Cx*. *aikenii* and *Culex* sp. in the Oriboca forest, Marituba (PA) in the state of Pará. In experimental studies with *Cx. quinquefasciatus*, the virus was identified in the salivary glands after four serial passages [26,27].

#### 4.3.4. Benfica Virus (BENV): *Orthobunyavirus benficaense*

The first isolation of BENV was performed in 1965 from brain and liver samples of newborn sentinel mice captured in the Utinga forest, Belém (PA), and by FC, HI and N tests BENV was classified as a member of the Capim group. It was also identified in samples of mosquitoes belonging to the *Culex* spp. genus collected in the Guamá Ecological Research Area (APEG) in Belém [26,27,38].

#### 4.3.5. BushBush Virus (BSBV): *Orthobunyavirus bushbushense*

BSBV was initially isolated from pools of female *Cx*. (*Aedinus*) *accelerans* collected in 1959 in Nariva Country, Trinidad. It is antigenically classified as the prototype of the BushBush complex, which is one of the five complexes that make up the Capim virus serogroup [26]. Reports from Karabatsos [27] and Hervé et al. [38] indicate its presence in *Culex* spp. mosquitoes collected from the Pará state of Brazil.

#### 4.3.6. Caraparu Virus (CARV): *Orthobunyavirus caraparuense*

CARV was first isolated in 1956 from serum and plasma samples collected from sentinel NHP *Cebus apella* captured in the forest of the Instituto Agronômico do Norte (IAN) in Belém. According to the CDC [26] and Karabatsos [27], it is antigenically related to the Apeú, Itaqui, Oriboca, and Murutucu viruses, and is part of the Caraparú virus complex. Pinheiro [48] proposes that the transmission cycle of the CARV in Brazil involves rodents, particularly from the *Proechimys* and *Oryzomys* genera, and *Culex* mosquitoes, notably the *Cx*. (*Mel*.) *vomerifer*, as the vectors.

Regarding the detection of CARV in *Culex* mosquitoes in Brazil, it has already been identified in *Culex* sp., *Cx*. *vomerifer*, and *Cx*. *portesi* collected in Belém (PA) [26,27,38].

#### 4.3.7. Capim Virus (CAPV): *Orthobunyavirus capimense*

CAPV was first isolated in 1958 from the liver and spleen samples of an adult marsupial (*Caluromys philander*) captured in the Utinga Forest, Belém (PA). It is now regarded as the prototype virus of the Capim serogroup, which consists of eight viruses [26,27]. It has also been identified in mosquitoes of the *Culex* genus, such as *Culex* sp., *Cules* sp. B1, *Cx*. *portesi*, *Cx*. pedroi, *Cx*. *coronator*, and *Cx*. *taeniopus* [26,27,38,85].

#### 4.3.8. Catu Virus (CATUV): *Orthobunyavirus catuense*

CATUV was first isolated from serum and plasma samples of a 17-year-old man who resided near the Oriboca forest in Marituba (PA). This individual experienced fever, headache, and myalgia for five days [27]. CTUV was categorized as part of the Guamá group and belonged to the Catu virus complex, which is one of five complexes in the Guamá serogroup [26,27].

CATUV has also been isolated from samples of mosquitoes, including *Culex* sp., *Cx*. *declarator*, and *Cx*. *portesi*, collected in Belém (PA). Experimental studies conducted with *Cx*. *portesi* showed that the virus was transmitted to a marsupial after 14 days of feeding on a viremic marsupial of the genus Zygodontomys [26,27].

#### 4.3.9. Cananeia Virus (CNAV): *Orthobunyavirus bertiogaense*

CNAV was isolated for the first time in 1976 from brain samples of newborn mice caught in the municipality of Cananéia (SP). Researchers analyzed the virus using different serologic methods and determined that CNAV is related to Bertigoa and Guaratuba viruses, as well as somewhat to the Mirim virus, all of which are part of the Guamá virus serogroup, to which CNAV also belongs [27]. Regarding isolation in mosquitoes of the *Culex* genus, CNAV was also isolated in the municipality of Cananéia (SP) from samples of *Cx*. (*Mel*.) *taeniopus* [27].

#### 4.3.10. Enseada Virus (ENSV): *Orthobunyavirus* enseadaense

ENSB was first isolated in 1976 in the municipality of Cananeia (SP) from a pool of female *Cx*. (*Mel*.) *taeniopus*. It was also detected in *Cx*. (*Mel*.) *epanastasis* collected in São Paulo [26,27].

#### 4.3.11. Guamá Virus (GMAV): *Orthobunyavirus guamaense*

GMAV was first isolated from serum and plasma samples of sentinel NHP *Cebus apella* captured in the Oriboca Forest, Marituba (PA) in 1955. This virus is considered the prototype of the Guama complex [26,27].

Travassos da Rosa et al. [85] reported the detection of GMAV in *Cx*. *portesi* and Causey et al. [32] pointed out that GMAV was isolated in 1959 from a pool of 97 *Cx*. (*Mel*.) sp. collected from the IAN forest, by the mouse viral isolation methodologies. Other *Culex* species, including *Cx*. *portesi*, *Cx*. (*Mel*.) *taeniopus*, and *Culex* sp. B17, have also tested positive for GMAV [26,27,56].

Toda and Shope [52] reported a study on vector competence conducted in 1964 in IAN forest in Belém. Mosquitoes were captured in traps containing chicken and rat bait, identified as the species *Cx*. (*Mel*.) *taeniopus* and kept in entomological cages in the forest. Newborn Swiss mice and their mothers were placed inside these cages as a blood meal source for 24 h. As a result, GMAV was identified in the blood of one mother.

#### 4.3.12. Guajará Virus (GJAV): *Orthobunyavirus guajaraense*

GJAV was initially isolated in 1959 from a Swiss mouse captured in IAN forest in Belém (PA). It is classified as a distinct virus type and belongs to the Guajará complex, which composes the Capim virus serogroup. It has been identified in *Culex* sp. and *Cx*. *portesi* in the state of Pará. In experimental studies conducted with the *Cx*. *quinquefasciatus*, the virus was detected in the salivary glands after four serial passages [26,27,38].

#### 4.3.13. Itaqui Virus (ITQV): *Orthobunyavirus oribocaense*

ITQV was isolated for the first time in 1959 from an adult female Swiss mouse captured in the IAN forest in Belém (PA) and is antigenically classified as a subtype of the Oriboca virus and a member of the Oriboca complex [26]. It was later identified in *Culex* spp. *Cx*. *portesi* and *Cx*. *vomerifer* mosquitoes collected in the state of Pará [26,27].

#### 4.3.14. Moju Virus (MOJUV): *Orthobunyavirus guamaense*

MOJUV was first isolated in 1959 from female *Cx*. (*Mel*.) spp. mosquitoes collected in the IAN forest in Belém (PA), and antigenically classified as a distinct virus belonging to the Guama virus complex. Was also detected in *Cx*. *portesi* and Cx. *vomerifer* in the State of Pará [26,27].

#### 4.3.15. Maguari Virus (MAGV): *Orthobunyavirus maguariense*

MAGV was isolated for the first time in pools of female mosquitoes containing several species, such as *Aedes serratus*, *Aedes scapularis*, *Aedes sexlineatus*, *Mansonia* sp. and *Psorophora ferox*, collected in the Utinga forest of Belém (PA) in 1957 [26,27]. The virus is considered a subtype of the Cache Valley virus and is linked with the Cache Valley and Tensaw viruses [26].

It was identified in samples of *Cx*. *taeniopus* collected in Belém (PA), and in experimental studies, the species *Cx*. *quinquefasciatus*, *Cx*. *nigripalpus*, and *Cx*. *corniger*, parentally inoculated, were capable of transmitting MAGV between the 7th and 23rd days of life [26].

#### 4.3.16. Mirim Virus (MIRV): *Orthobunyavirus mirimense*

MIRV was initially found in serum and plasma samples from female NHP *Cebus apella* captured in the IAN forest in Belém (PA) in 1957. The virus was also identified in samples of *Cx*. *taeniopus* and *Cx*. (*Mel*.) sp. collected in the state of Pará. Experimental studies illustrated that naturally infected *Cx*. *taeniopus* mosquitoes transmitted the virus to mice [26,27].

#### 4.3.17. Marituba Virus (MTBV): *Orthobunyavirus maritubaense*

MTBV was first isolated in serum and plasma samples from male NHP *Cebus apella* captured in the Oriboca Forest, Marituba (PA) in 1954. Classified as a distinct viral type, the Marutucu and Restan viruses are considered subtypes of the Marituba virus. Additionally, it was identified in *Cx*. *aikenii*, *Cx*. (*Mel*.) sp., and *Cx*. *portesi* collected in Belém (PA) [27].

#### 4.3.18. Murutucu Virus (MURV): *Orthobunyavirus maritubaense*

MURV was first isolated in 1955 from serum and plasma samples from NHP *Cebus apella* captured in the IAN forest in Belém (PA). It belongs to the Marituba virus subtype and has been detected in several types of mosquitoes, including *Culex* sp. B1, B7, B19, *Cx*. *aikenii*, *Cx*. *caudelli*, and *Cx*. *portesi* in the state of Pará [26,27,56].

#### 4.3.19. Nepuyo Virus (NEPV): *Orthobunyavirus nepuyoi*

NEPV was first isolated in 1957 in Neriva-Mayaro, Trinidad, from a pool of *Cx*. (*Aedinus*) *accelerans*. It belongs to the Marituba virus complex and was later identified in *Culex* sp. samples collected in the state of Pará, Brazil. Furthermore, experiments performed on parentally infected *Cx*. *quinquefasciatus* revealed that the virus could be maintained in the salivary glands for two consecutive rounds of infection [26,27].

#### 4.3.20. Oriboca Virus (ORIV): *Orthobunyavirus oribocaense*

ORIV was first isolated from serum and plasma samples of *Cebus apella* captured in the Oriboca Forest, Marituba (PA) in 1954, and is classified as a virus belonging to the Oriboca virus complex, and it should be noted that Itaqui virus is considered a subtype of Oriboca virus [26,27].

It was detected in samples of *Culex* spp. and *Cx*. *portesi* collected in Belém (PA) [27], and Toda and Shope [52] conducted a study with about 2860 female *Cx*. (*Mel*.) spp. released in an entomological cage and exposed to baby mice as a source of blood feeding. From this study, ORIV was isolated from one exposed baby mouse.

#### 4.3.21. Oropouche Virus (OROV): *Orthobunyavirus oropoucheense*

OROV is a negative-sense, single-stranded RNA arbovirus first isolated in 1955 in Trinidad from a human blood sample with a benign febrile illness [47,86]. It is maintained in nature by wild cycles involving wild mammals such as sloths and NHP as reservoirs and amplification hosts, and dipterans as vectors such as the species *Culicoides paraensis*. However, other arthropods, such as *Coquillettidia venezuelensis*, *Aedes serratus*, and *Cx. quinquefasciatus*, can also be secondary vectors [21].

During epidemics in cities in the state of Pará such as Belém, Bragança, Itupiranga and Santarém in 1975, OROV was isolated from samples of *Cx*. *quinquefasciatus*. In Bragança, the virus was isolated from a pool containing engorged females. In Belém was detected in two pools also of *Cx*. *quinquefasciatus*. However, no virus was isolated from samples of *Cx*. *quinquefasciatus* from the cities of Itupiranga and Santarém, being identified only in samples of *Culicoides paraensis* [27,47,59].

Ferreira et al. [36] also detected 20 pools of *Cx*. *quinquefasciatus* mosquitoes collected in the city of Cuiabá (MT) positive for OROV by viral isolation.

Vector competence studies have been conducted to determine the potential vector competence of *Culex* for OROV. Hoch et al. [59] utilized adult females of *Cx*. *quinquefasciatus* captured at various locations in Belém (PA) from 1975 to 1978. Three experiments were conducted on the F1 generation. The female subjects were exposed to a viremic hamster, and Syrian golden hamsters aged 21 to 23 days were used for virus transmission assessment. The study indicated that none of the hamsters that were exposed to infected mosquitoes manifested signs of virus infection, nor were any OROV antibodies detected in their blood samples.

Studies were also conducted on *Cx*. *quinquefasciatus* mosquitoes from the city of Belo Horizonte (MG), which demonstrated resistance to oral infection by OROV. The females were exposed to infected AG129 mice and tested individually on the 7th and 14th dpi after oral infection, with negative results. The species was evaluated for intrathoracic infection using intrathoracic nanoinjection and was also evaluated at 7 and 14 dpi, with a 100% infection rate observed at both dpi’s evaluated, as well as the dissemination of OROV in the midgut after intrathoracic infection, observing that the virus is not able to overcome the infection barriers of the midgut [21].

#### 4.3.22. Pacora Virus (PACV): *Orthobunyavirus pacoraense*

PACV was first isolated in 1958 from samples of female *Culex dunni* mosquitoes collected in the Pacora district of Panama [27,51]. The transmission cycle of PACV is thought to occur between birds as amplification hosts and *Culex* mosquitoes as primary vectors [51].

In 1992, two strains of PACV virus were isolated from the analysis of 36 pools of *Culex* spp. mosquitoes at the Amazon Biopark, formerly known as the Zoobotanical Garden, located on the Macapá-Santana highway in Macapá [51].

#### 4.3.23. Triniti Virus (TNTV): *Orthobunyavirus trinitiense*

TNTV was first isolated in 1955 from a pool of 37 mosquitoes of the genus *Trichoprosopon* collected in Saint (St.) George’s County, Trinidad [26]. Even though it has not yet been classified into any antigenic group, it exhibited a weak crossover with the Aruac and Ieri viruses when examined via the CF test [26]. Segura et al. [49] reported the discovery of TNTV in specimens of *Cx*. (*Mel*.) sp. collected in 2001 from the Caxiuanã National Forest (PA).

#### 4.3.24. Tucunduba Virus (TUCV): *Orthobunyavirus wyeomyiae*

TUCV is a single-stranded RNA virus that belongs to the Wyeomyia complex [87]. Monteiro [43] isolated a TUCV strain from a pool of *Cx*. *coronator* mosquitoes collected in the Igarapé Gelado Environmental Protection Area, located 60 km from Parauapebas (PA). In 2001, Segura et al. [49] reported the isolation of TUCV from *Cx*. *declarator* mosquitoes collected in Altamira (PA). Isolation was performed by intracerebral inoculation of 0.02 mL suspension of macerates from lots of mosquitoes into Swiss baby mice.

#### 4.3.25. Turlok Virus (TURV): *Orthobunyavirus turlockense*

TURV was first isolated in 1954 from female *Culex tarsalis* mosquitoes collected near Sutter City, California (USA). It belongs to the Turlok complex, one of the three complexes comprising the Turlok serogroup. The virus is related to the Umbre virus, discovered in India, and the M’Poko virus, identified in Central Africa. In Brazil, it was detected in *Culex portesi* mosquitoes in Belém (PA) [26,27].

### 4.4. Rhabdoviridae: Hapavirus

#### Mosqueiro Virus (MQOV): *Hapavirus mosqueiro*

MQOV was first isolated in 1970 from a pool of engorged *Cx*. *portesi* females collected at the Instituto de Pesquisas e Experimentação Agropecuárias do Norte-IPEAN, now EMBRAPA-Amazônia Oriental. The MQOV was found to be related to the Hart Park and Kamese viruses, as evidenced by the results of the CF test [26,27].

### 4.5. Phenuiviridae: Phlebovirus

#### 4.5.1. Itaporanga Phlebovirus (ITPV): *Phlebovirus itaporangaense*

ITPV was first isolated in 1962 from a brain sample of a Swiss baby mouse collected after its death in the city of Itaporanga (PA). It has also been identified in *Cx*. *caudelii* collected in Belém (PA) and in *Culex* spp. collected in Belém (PA) and Amapá [26,27].

#### 4.5.2. Icoaraci Virus (ICOV): *Phlebovirus icoaraciense*

ICOV was first isolated in 1960 from samples of liver, kidney, heart, and spleen from a rodent of unidentified species. The rodent was captured at the IAN forest, EMBRAPA-Amazônia Oriental, Belém (PA). Additionally, it was discovered in *Culex* sp. mosquitoes collected in the state of São Paulo [26,27].

### 4.6. Paramyxoviridae: Orthorubulavirus

#### Mapuera Virus (MapV): *Orthorubulavirus mapueraense*

MapV was isolated for the first time in 1979 by analyzing the salivary glands of *Sturnira lillium* bats captured in the quilombola area of Cachoeira Porteira, in Oriximina (PA). The virus was also present in male *Culex* spp. mosquitoes collected from the same area [26,27].

### 4.7. Sedoreoviridae: Orbivirus

#### Jacareacanga Virus (JACV): *Corriparta virus*

JACV virus was isolated in 1975 from a pool of 25 *Culex* (*Mel*.) sp. females collected at km 212 (Flexal) of the Transamazon highway, between the cities of Itaituba and Jacareacanga [26,27].

### 4.8. Ungrouped

#### Para Virus (PARAV)

PARAV is not currently associated with any viral family and was first isolated in 1975 from a sample of the brain and liver of a newborn Swiss mouse captured in the Guamá Ecological Research Area (APEG) in Belém (PA) and was also detected in *Cx*. (*Mel*.) *ocossa* in the municipality of Uruaçu, Goiás [26,27].

## 5. Conclusions

In Brazil, the *Culex* genus is recognized as the primary vector of *Wuchereria bancrofti*, the etiological agent of lymphatic filariasis, but worldwide they are also important vectors of arboviruses such as WNV and SLEV. Therefore, the study of the genus *Culex* and its role as arbovirus vector in Brazil is crucial to understand the transmission cycles and viral circulation dynamics in the country, and to implement actions aimed at reducing infections caused by viruses transmitted by these arthropods. Due to their wide geographical range, their adaptability to different environments and their bionomics, *Culex* mosquitoes are susceptible to different viruses, playing a pivotal role in the generation of evolutionary processes between pathogen and host, making them potential vectors of arboviruses.

We emphasize the importance of entomological and virological research on mosquitoes collected in the wild and in urban areas to identify new viruses and natural infections of new vectors. However, it should be noted that the identification of infected mosquitoes in the field does not directly establish natural infections or their role as vectors in the transmission of these viruses. The virus detected may be blood residues from feeding on a viremic host. Thus, to determine the participation of these mosquitoes in transmission cycles more accurately, it is necessary to conduct studies on the virus’s infectivity in the arthropod’s tissues and its ability to transmit through blood meal.

## Figures and Tables

**Table 1:** Summary of the articles that made up the integrative review and the viruses detected according to the specimen analyzed.

Citation	Year	Virus	Species
Araújo et al. [29]	2019	Rocio virus	*Culex* (*Melanoconion*) *portesi*
		Ilhéus virus	
		Bussuquara virus	
Ayres et al. [30]	2019	Zika virus	*Culex quinquefasciatus*
Barrio-Nuevo et al. [31]	2020	Dengue virus (DENV 2)	*Culex* spp.
			*Culex vaxus*
Causey et al. [32]	1961	Bussuquara virus	*Culex* (*Mel.*) sp.
		Guamá virus	
Causey et al. [33]	1963	Aurá virus	*Culex* (*Mel.*) sp.
Cruz et al. [34]	2020	Chikungunya virus	*Cx. quinquefasciatus*
Cunha et al. [35]	2020	Ilhéus virus	*Culex* sp.
Ferreira et al. [36]	2020	Chikungunya virus Zika virus Mayaro virus Oropouche virus	*Cx. quinquefasciatus*
Guedes et al. [37]	2017	Zika virus	*Cx. quinquefasciatus*
Hervé et al. [38]	1986	Acará virus	*Culex* sp.
		Ananindeua virus	*Cx. portesi*
			*Cx. pedroi*
			*Cx. aikenii*
			*Culex* sp.
		Benfica virus	*Culex* sp.
		Bushbush virus	*Culex* sp.
		Bussucuara virus	*Culex* sp.
			*Cx declarator*
			*Cx.* (*Mel.*) sp.
			*Cx. pedroi*
		Caraparu virus	*Culex* sp.
			*Cx. coronator*
			*Cx.* (*Mel.*) sp.
			*Cx portesi*
			*Cx. vomerifer*
			*Cx. spissipes*
		Capim virus	*Cx. portesi*
			*Cx. pedroi*
			*Cx. coronator*
			*Culex* sp.
		Catú virus	*Cx. portesi*
			*Cx.* (*Mel.*) sp.
			*Cx. declarator*
			*Culex* sp.
		Guajará virus	*Culex* sp.
		Guamá virus	*Cx. portesi*
			*Cx.* (*Mel.*) sp.
			*Cx.* sp*issipes*
			*Cx. pedroi*
			*Culex* sp.
		Itaporanga phlebovirus	*Cx.* (*Mel.*) sp.
			*Culex* sp.
		Itaqui virus	*Cx. portesi*
			*Cx. pedroi*
			*Cx* sp*issipes*
			*Cx. vomerifer*
			*Culex* sp.
		Mucambo virus	*Cx. portesi*
			*Cx.* (*Mel.*) sp.
			*Cx.* (*Cux.*) sp.
		Moju virus	*Cx.* (*Mel.*) sp.
			*Cx. vomerifer*
			*Culex* sp.
		Murutucú virus	*Cx. portesi*
			*Cx.* (*Mel*) sp.
			*Culex* sp. B27
		Nepuyo virus	*Culex* sp.
		Oriboca virus	*Cx. portesi*
			*Cx. pedroi*
			*Cx.* sp*issipes*
			*Cx.* (*Mel.*) sp.
			*Culex* sp.
Heinen et al. [39]	2015	St. Loius encephalitis virus	*Cx. quinquefasciatus*
Krokovsky et al. [40]	2022	Chikungunya virus	*Cx. quinquefasciatus*
		Zika virus	
		Dengue virus (DENV2 e 3)	
Krokovsky et al. [41]	2022	Zika virus	*Cx. quinquefasciatus*
Moraes et al. [42]	2019	Dengue virus (DENV4)	*Cx. quinquefasciatus*
Monteiro [43]	2009	Tucunduba virus	*Cx. coronator*
Nunes Neto et al. [44]	2023	West Nile virus	*Culex* (*Mel.*) sp.
Neves et al. [45]	2022	Chikungunya virus	*Cx. quinquefasciatus*
		Mayaro virus	
		Zika virus	
Paiva et al. [46]	2020	Zika virus	*Cx. quinquefasciatus*
Pinheiro et al. [47]	1981	Oropouche virus	*Cx. quinquefasciatus*
Pinheiro et al. [48]	1982	Bussuquara virus	*Culex* sp. B1
			*Culex* sp.
		Capim virus	*Culex* sp. B1
		St. Loius encephalitis virus	*Cx. coronator*
			*Cx. declarator*
		Mucambo virus	*Cx.* (*Mel.*) *portesi*
		Oriboca virus	
		Catú virus	
		Guamá virus	
		Itaqui virus	*Cx.* (*Mel.*) *portesi* *Cx.* (*Mel.*) *vomerifer*
		Caraparú virus	*Cx.* (*Mel.*) *vomerifer*
		Ananindeua virus	*Cx.* (*Mel*.) sp.
			*Cx.* (*Mel.*) *portesi*
			*Cx. taeniopus*
			*Cx. vomerifer*
			*Culex* sp. B19
			*Culex* sp. B27
Segura et al. [49]	2004	Triniti virus	*Cx.* (*Mel.*) sp.
		Tucunduba virus	*Cx. declarator*
		St. Louis encephalitis virus	
		Eastern equine encephalitis virus	*Cx.* (*Mel.*) sp.
Serra et al. [50]	2016	Dengue virus (DENV4)	*Cx. quinquefasciatus*
			*Cx. bidens/interfor*
		Mayaro virus	*Cx. quinquefasciatus*
		St. Loius encephalitis virus	*Cx. quinquefasciatus*
Souto et al. [51]	1996	Pacora virus	*Cx.* spp.
Toda; Shope [52]	1965	Guamá virus	*Cx.* (*Mel.*) *taeniopus*
Travassos da Rosa et al. [53]	1982	St. Loius encephalitis virus	*Cx. declarator*
			*Cx. coronator*
			*Cx.* (*Mel.*) *spissipes*
			*Cx.* (*Mel.*) *portesi*
			*Culex* sp. (B19)
Tschá et al. [54]	2019	Caaingua virus	*Culex* spp.
Vieira et al. [55]	2019	Ilhéus virus	*Cx. coronator*
			*Cx.* (*Mel.*) sp.
Woodall [56]	1967	Eastern Equine Encephalitis Virus	*Cx.* (*Mel*) *taeniopus*
			*Cules* spp.
			*Culex* sp. (B1, B9)
			*Cx.* (*Mel*) sp.
			*Cx.* (*Mel.*) sp*issipes*
		Aura virus	*Cx.* (*Mel.*) sp.
		Mayaro virus	*Culex* spp.
		Mucambo virus	*Culex* spp.
			*Culex* sp. (B7, B9, B19)
			*Cx.* (*Mel.*) sp.
		Bussuquara virus	*Culex* sp.
			*Culex* sp. (B1, B7)
			*Cx.* (*Mel.*) sp.
			*Cx.* (*Mel*) *taeniopus*
		St. Loius encephalitis virus	*Cx.* (*Cux.*) *declarator*
		Apeú virus	*Culex* sp. (B19)
		Caraparú virus	*Culex* spp.
			*Culex* sp. (B7, B9)
			*Cx.* (*Mel.*) *caudelli*
			*Cx* (*Mel.*) *spissipes*
		Nepuyo virus	*Culex* spp.
		Itaquí virus	*Culex* spp.
			*Culex* sp. (B7, B9, B17, B19)
			*Cx.* (*Mel.*) *spissipes*
		Oriboca virus	*Culex* spp.
			*Culex* sp. B9
			*Cx.* (*Mel.*) sp.
			*Cx.* (*Mel.*) *caudelli*
			*Cx* (*Mel.*) *spissipes*
		Catú virus	*Culex* sp. B9
			*Cx.* (*Cux.*) *declarator*
		Guamá virus	*Culex* spp.
			*Culex* sp. (B1, B7, B9, B17, B19)
			*Cx.* (*Mel.*) sp.
			*Cx* (*Mel.*) *spissipes*
			*Cx.* (*Mel*) *taeniopus*
		Moju virus	*Culex* spp.
			*Culex* sp. (B7, B9)
			*Cx.* (*Mel.*) sp.
		Capim virus	*Culex* spp.
			*Culex* sp. B1
		Guajará virus	*Culex* spp.
			*Culex* sp. (B1, B9)
		Bushbush virus	*Culex* spp.
		Mirim virus	*Cx.* (*Mel*) *taeniopus*
		Itaporanga phlebovirus	*Culex* spp.
			*Cx.* (*Mel.*) *caudelli*

Subtitle: “sp.” indicates a group of specimens belonging to the same species, but it was not possible to identify the species; “spp.” indicates a group of mosquitoes belonging to the same genus; “B1, B7, B9, B17 and B19” indicate unidentified morphospecies of the genus *Culex* collected in the city of Belém (PA).

**Table 2:** Experimental studies on the vector competence of the *Culex quinquefasciatus* species for different arboviruses.

Citation	Year	Virus	Colony Location	Methodology	Summary of Results
Fernandes et al. [57]	2016	ZIKV	Rio de Janeiro (RJ)	Oral infection with artificial feeder	Refractory to ZIKV
Fernandes et al. [58]	2017	ZIKV	Recife (PE)/Rio de Janeiro (RJ)	Oral infection with artificial feeder	Refractory to ZIKV
Guedes et al. [37]	2017	ZIKV	Recife (PE)	Oral infection with artificial feeder	ZIKV susceptible colony with viral detection in saliva
Hoch et al. [59]	1987	OROV	Belém (PA)	Viremic and virgin hamsters	Low transmission efficiency
Krokovsky et al. [60]	2023	MAYV	Recife (PE)	Oral infection with petri dish	Susceptible to the virus, but with inefficient transmission.
Mendonça et al. [21]	2021	OROV	Belo Horizonte (MG)	Oral infection with artificial feeder and nanoinjection	Refractory in oral infection and susceptible when nanoinjected into the chest
Pereira et al. [61]	2020	MAYV	Belo Horizonte (MG)	Oral infection with artificial feeder	Low infection rate and inefficient transmission
Reis et al. [62]	2023	WNV	Ananindeua (PA)	Oral infection with artificial feeder	Susceptible, with high rates of infection, dissemination, and transmission

**Table 3:** Arbovirus detected by viral isolation and molecular biology in *Culex* mosquitoes collected in Brazil, 2023.

Family	Genus	Viral Species	Virus Name	Abbreviation
*Togaviridae*	*Alphavirus*	*Aura virus*	Aura virus	AURAV
		*Caaingua virus*	Caaingua virus	CAAV
		*Chikungunya virus*	Chikungunya virus	CHIKV
		*Eastern equine encephalitis virus*	Eastern equine encephalitis virus	EEEV
		*Mayaro virus*	Mayaro virus	MAYV
		*Mucambo virus*	Mucambo virus	MUCV
*Flaviviridae*	*Orthoflavivirus*	*Orthoflavivirus aroaense*	Bussuquara virus	BSQV
		*Orthoflavivirus denguei*	Dengue virus	DENV
		*Orthoflavivirus ilheusense*	Ilhéus virus	ILHV
		*Orthoflavivirus ilheusense*	Rocio virus	ROCV
		*Orthoflavivirus louisense*	St. Louis encephalitis virus	SLEV
		*Orthoflavivirus nilense*	West Nile virus	WNV
		*Orthoflavivirus zikaense*	Zika virus	ZIKV
*Peribunyaviridae*	*Orthobunyavirus*	*Orthobunyavirus ananinde* *uaens* *e*	Ananindeua virus	ANUV
		*Orthobunyavirus acaraense*	Acará virus	ACAV
		*Orthobunyavirus apeuense*	Apeú virus	APEUV
		*Orthobunyavirus benficaense*	Benfica virus	BENV
		*Orthobunyavirus bushbushense*	Bushbush virus	BSBV
		*Orthobunyavirus caraparuense*	Caraparú virus	CARV
		*Orthobunyavirus capimense*	Capim virus	CAPV
		*Orthobunyavirus catuense*	Catú virus	CATUV
		*Orthobunyavirus bertiogaense*	Cananéia virus	CNAV
		*Orthobunyavirus enseadaense*	Enseada virus	ENSV
		*Orthobunyavirus guamaense*	Guamá virus	GMAV
		*Orthobunyavirus guajaraense*	Guajará virus	GJAV
		*Orthobunyavirus oribocaense*	Itaquí virus	ITQV
		*Orthobunyavirus guamaense*	Moju virus	MOJUV
		*Orthobunyavirus maguariense*	Maguari virus	MAGV
		*Orthobunyavirus mirimense*	Mirim virus	MIRV
		*Orthobunyavirus maritubaense*	Marituba virus	MTBV
		*Orthobunyavirus maritubaense*	Murutucú virus	MURV
		*Orthobunyavirus nepuyoi*	Nepuyo virus	NEPV
		*Orthobunyavirus oribocaense*	Oriboca virus	ORIV
		*Orthobunyavirus oropoucheense*	Oropouche virus	OROV
		*Orthobunyavirus pacoraense*	Pacora virus	PCAV
		*Orthobunyavirus trinitiense*	Triniti virus	TNTV
		*Orthobunyavirus wyeomyiae*	Tucunduba virus	TUCV
		*Orthobunyavirus turlockense*	Turlock virus	TURV
*Rhabdoviridae*	*Hapavirus*	*Hapavirus mosqueiro*	Mosqueiro virus	MQOV
*Phenuiviridae*	*Phlebovirus*	*Phlebovirus itaporangaense*	Itaporanga phlebovirus	ITPV
		*Phlebovirus icoaraciense*	Icoaraci virus	ICOV
*Paramyxoviridae*	*Orthorubulavirus*	*Orthorubulavirus mapueraense*	Mapuera virus	MapV
*Sedoreoviridae*	*Orbivirus*	*Corriparta virus*	Jacareacanga virus	JACV
*Ungrouped*	*Ungrouped*	*Para virus*	Pará virus	PARAV

## Data Availability

The data presented in this study are available in the article.
